# Trends in Primary Mental Health Care Service Use and Subsequent Self-Harm in Western Sydney Australia: Policy and Workforce Implications

**DOI:** 10.3390/ijerph19063382

**Published:** 2022-03-13

**Authors:** Sithum Munasinghe, Andrew Page, Sandro Sperandei, Pankaj Gaur, Shahana Ferdousi, Haider Mannan, Vlasios Brakoulias

**Affiliations:** 1 Translational Health Research Institute, Campbelltown Campus, Western Sydney University, Locked Bag 1797, Penrith, NSW 2751, Australia; a.page@westernsydney.edu.au (A.P.); s.sperandei@westernsydney.edu.au (S.S.); h.mannan@westernsydney.edu.au (H.M.); vlasios.brakoulias@health.nsw.gov.au (V.B.); 2 Western Sydney Primary Health Network, Level 1/85 Flushcombe Road, Blacktown, NSW 2148, Australia; shahana.ferdousi@wentwest.com.au; 3 Western Sydney Local Health District, Integrated and Community Health Department, 5 Fleet Street, North Parramatta, NSW 2151, Australia; pankaj.gaur@health.nsw.gov.au; 4 Western Sydney Local Health District, Mental Health Service, 1-11 Hainsworth Street, Westmead, NSW 2145, Australia

**Keywords:** primary mental health care, psychological treatments, self-injurious behavior, mental health policy

## Abstract

Background: This study investigated the trends in primary mental health care (PMHC) service use and hospital-treated self-harm in Western Sydney (Australia). Methods: A data linkage study and descriptive ecological study of PMHC referrals investigated the trends in referrals, treatment attendance, hospital-treated self-harm, and health care practitioners (HCPs) for the period of 2013−2018 (*n* = 19,437). Results: There was a substantial increase in referrals from 2016. The majority of referrals were females (60.9%), those aged <45 years (71.3%), and those presenting with anxiety or affective disorders (78.9%). Referrals of those at risk of suicide increased from 9.7% in 2013 to 17.8% in 2018. There were 264 (2.2%) cases of subsequent hospital-treated self-harm, with higher rates among those at risk of suicide and those who attended <6 sessions. The number of HCPs per referral also increased from 2013, as did waiting times for treatment initiation. Conclusion: Individuals presenting to PMHC services at risk of suicide, and who subsequently presented to a hospital setting following self-harm, were more likely to either not attend services following a referral or to attend fewer services. This trend occurred in the context of an increase in the number of clients per HCP, suggesting workforce capacity has not kept pace with demand.

## 1. Introduction

The Australian government has made significant investments in mental health services over the last two decades by introducing initiatives for those who experience mental disorders to receive mental health services at no cost or low cost [1,2]. These services were widened under the ‘Better Access’ initiative in 2007, which allowed Medicare rebates for those receiving mental health treatments via psychologists, psychiatrists, or general practitioners [3]. Additional reforms followed the transition of government-funded primary mental health care (PMHC) services from Medicare Locals to Primary Health Networks (PHNs) in 2016 and recommendations from the National Mental Health Commission [4] relating to the local commissioning of services in order to reflect local priorities. Following these policy reforms, a stepped care model of PMHC also incorporated the provision of low-intensity interventions for those experiencing or at risk of mild mental health conditions, as well as interventions that are more intensive for those experiencing severe and complex mental health conditions [5]. Overall, more than AUD 10.5 billion was invested by the Australian government for all primary and acute care mental health services over the 2018–2019 financial year [6].

Despite increased investments in mental health services, the prevalence of mental disorders and the incidents of self-harm and suicide have not changed substantially in Australia [7,8]. Recent studies have suggested that the increased prevalence of mental disorders may not be due to the increased awareness of mental health conditions and wider access to mental health services [9], or increases in risk factors for common mental disorders [8]. Instead, inadequate treatments due to poor treatment engagement [10], inequalities in target groups in terms of mental health needs [11], poor outcome monitoring, and inadequate use of routinely collected data to monitor the effectiveness of treatments [12] are likely the important and preventable determinants of mental disorders despite wider access to mental health care.

Studies that investigate primary mental health services in terms of subsequent self-harm presentations to hospital settings are sparse in Australia, especially in local contexts. Given that local approaches to mental health services have been recommended by recent policy reforms [4], studies are needed to timely inform service improvements by acknowledging the available resources and capacity. Accordingly, this study investigates (i) primary mental health care service use, (ii) workforce capacity, and (iii) subsequent hospital-treated self-harm in the geographic catchment of Western Sydney (Australia). The findings of the current study will help to inform the key areas that need service and policy responses to improve the effectiveness of routine mental health services to better mental health outcomes.

## 2. Materials and Methods

### 2.1. Study Catchment

This study was based on the population catchment of the Western Sydney Primary Health Network (WSPHN), which covers four local government areas, namely, Cumberland, Blacktown, Parramatta, and Hills Shire, in the greater Western Sydney region (Australia). The WSPHN population catchment is a population of approximately one million [13], and it is characterized by substantial socioeconomic, geographic, and demographic diversity, with approximately 40% of the population from culturally and linguistically diverse backgrounds [14].

### 2.2. Study Participants

This study included all clients aged 5 years or over who accessed PMHC services (see below) over the six-year period from 1 January 2013 to 31 December 2018 (*n* = 16,268). Clients aged 18 years or over (*n* = 9924) were individually linked (see below) to hospital or emergency department (ED) data.

### 2.3. Primary Mental Health Care Services

PMHC services were established to provide access to mental health services for those who otherwise would have no, or low, access to mental health treatments [15]. Current PMHC services focus on six key areas, namely, (i) ‘*low intensity psychological interventions for those who experience, or at risk of, mild mental health conditions*’; (ii) ‘*psychological therapies delivered by mental health professionals for underserviced groups*’; (iii) ‘*early interventions for children and young people with, or at risk of, mental health conditions*’; (iv) ‘*treatments for those with severe and complex mental health conditions*’; (v) ‘*Aboriginal and Torres Strait Islander mental health services*’; and (vi) ‘*suicide prevention services for those who have attempted suicide, or present to a primary care setting in suicidal crisis*’ [16].

### 2.4. Data Sources

Data for PMHC services were extracted from two separate data sources to establish trends in PMHC services over time: (i) the PMHC Minimum Data Set (MDS) for the period of July 2016–December 2018 and (ii) the Access to Allied Psychological Services (ATAPS) MDS for the period of January 2013–July 2018. The PMHC MDS comprises information relating to clients’ sociodemographic, socioeconomic, and diagnostic information; referrer and service provider characteristics; and service session information, such as type of psychological intervention, mode of contact, and session duration (a full list of data items can be found in [16]). Following the establishment of the PMHC MDS, all ATAPS services were subsumed within the PMHC MDS from July 2017, and the ATAPS MDS was suspended in August 2018. Guidelines are available to facilitate the combination of these two different data sources [16].

In addition to the PMHC data, this study also used acute care data held by the Western Sydney Local Health District (WSLHD) (a district that corresponds to the geographic catchment of WSPHN). Acute care data included both ED and hospital-admitted presentations of patients who presented to Auburn, Blacktown, Cumberland, Mount Druitt, or Westmead Hospital settings in the greater Western Sydney region (Australia). PMHC data were also individually linked (see below) to acute care data to identify clients who presented to a hospital setting following self-harm for the period of 2011–2020.

The Index of Relative Socioeconomic Disadvantage (IRSD) of suburbs in the Western Sydney PHN catchment was used to derive the area-level socioeconomic status of clients, obtained from census years 2011 and 2016 [17], with intercensal IRSD scores estimated by linear interpolation.

### 2.5. Data Linkage

The current study employed probabilistic data linkage algorithms to link PMHC data with acute care data using individuals’ identifiable information, such as first name, last name, date of birth, and residential information, in order to link records between two data sources (described in detail in the Supplementary Materials). Individual identifiable information was not available for 2277 clients, and they were excluded from the linkage study (described in detail in the Supplementary Materials). Linkage quality was assessed including a clerical review (Table S1) and presented using sensitivity and specificity, with the sensitivity and specificity of the linkage being 98.37% and 95.74%, respectively (Table S2).

### 2.6. Study Factors

A series of sociodemographic, diagnostic, referral-, and service-level variables were included in the study. Sociodemographic information included gender (‘male’, ‘female’), age in years (‘5–17’, ‘18–24’, ‘25–44’, ‘45–64’, ‘ ≥65’), and socioeconomic status defined in terms of IRSD (postcodes of usual residence are categorized into population quintiles from ‘most deprived’ to ‘least deprived’).

Diagnoses in the current study were categorized into the following five categories, reflecting high prevalence mental disorders: anxiety disorder, affective disorder, anxiety and affective disorder, substance use disorder, and other. The substance use disorder category comprised clients presenting with either a primary or comorbid substance use diagnosis. Suicide risk (‘yes’ or ‘no’) in the PMHC MDS was defined as ‘yes’ if the referral was from the ED or hospital following self-harm, or if clients presented to a GP following self-harm (but not presenting to a hospital setting) or with strong suicidal ideation. Current medication use (‘yes’ or ‘no’) related to a current prescription for any antidepressants, antipsychotics, anxiolytics, psychostimulants and nootropics, or hypnotics and sedatives.

Service level variables included the number of treatment sessions attended (‘0’, ‘1–2’, ‘3–5’, ‘6’, ‘7–11’, ‘12 or +’) and waiting time for first treatment session (‘within 1 week’, ‘1–2 weeks’, ‘2–4 weeks’, ‘4+ weeks’). Workforce capacity included the number of health care practitioners (HCPs) and clients per HCP each year. HCPs included psychologists, social workers, occupational therapists, general practitioners, psychiatrists, and other medical and allied health care providers.

Cases of hospital-treated self-harm were identified based on International Classification of Diseases, version 10 (ICD–10), codes X60–X84 for intentional self-harm and Y10–Y34 for self-harm, with undetermined intent for hospital admission data. Systematized Nomenclature of Medicine (SNOMED) diagnosis and the ‘Presenting Problem text’ fields were used to identify cases presented to ED (Table S3), based on keywords relating to self-harm (see Supplementary Materials). This study included any subsequent self-harm presentation within 12 months following the last PMHC service contact or referral date (for those who never attended at least one treatment session).

### 2.7. Data Analysis

Descriptive analyses were conducted to examine trends in (i) client presentations to PMHC services and (ii) subsequent hospital-treated self-harm within 12 months, stratified by gender, age group, risk of suicide (as indicated in the PMHC MDS), and year. While many clients accessed PMHC services more than once during the six-year period, a single referral was used as the unit of analysis. Two referrals were considered as a single referral if the difference between the last session date of the first referral and the referral date of the second referral was less than 30 days. This is because (i) some clients may continue services under a ‘no’ suicide risk referral following an initial referral for suicide risk and (ii) a new referral is required if the client needs to continue services for more than one year, as the previous referral expires after one year for some service types. For example, diagnosis, medication use, focus of treatments, and other referral-level characteristics may change for some clients who remain engaged with services for 12 months, and a new referral, including updated information, is required to continue services for more than one year. Combining referrals in this way provided the total number of continuously attended treatment sessions during a particular period and enabled the definition of the key indicator of number of sessions per referral. Data analysis was conducted using Stata16.1 (Stata Corp, 4905 Lakeway Drive, College Station, Texas 77845, USA).

## 3. Results

**Figure 1:**
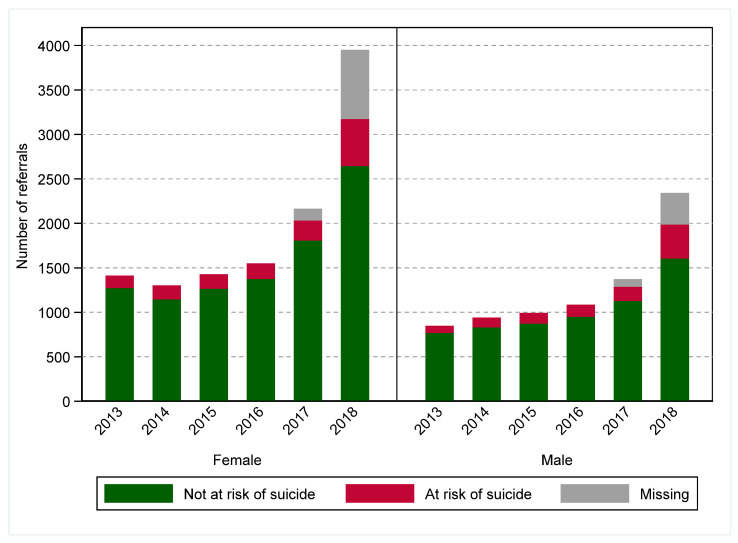
Number of referrals to primary mental health care (PMHC) services by gender, Western Sydney Primary Health Network, 2013–2018. Referrals with a missing suicide risk flag were considered as suicide referrals for those who received suicide-prevention-specific services (there were 222 referrals with missing suicide flags, which were replaced due to being at risk of suicide).

There were 19,437 referrals to PMHC services during the six-year period (Table 1), with a dramatic increase in referrals in 2017 and 2018 following the transition of the PMHC services from Medicare Locals to PHNs in 2016 (Figure 1). The majority of the referrals were for females (60.9%), younger age groups (<45 years) (71.3%), and those presenting with anxiety or affective disorders (78.8%) (Table 1). Nearly 13% of referrals were for those at risk of suicide, and a higher proportion of the referrals for those at risk of suicide were received in 2018 (17.8%) compared to previous years (Table 1). The proportion of those using any psychotropic medication also consistently increased from 29.6% in 2013 to 49.4% over the study period.

**Figure 2:**
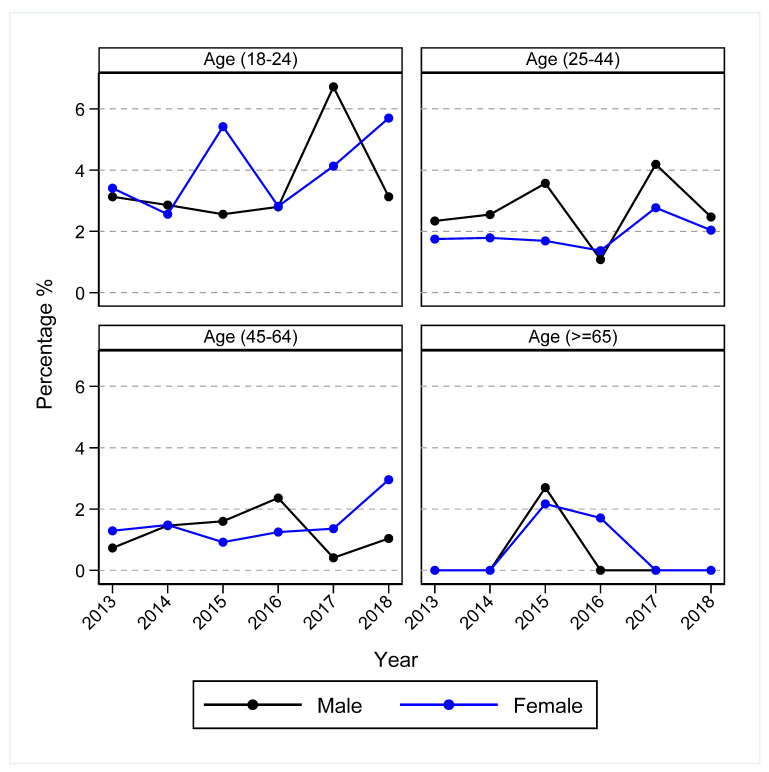
Trends in hospital-treated self-harm within 12 months of last service contact by sex and age group, Western Sydney Primary Health Network, 2013–2018. Any hospital treated self-harm presentation (including cases of undetermined intent) within 12-months of the last service contact (or referral date for referrals that did not result in at least one follow-up treatment session) was presented as a proportion of primary mental health care service referrals in each year.

**Figure 3:**
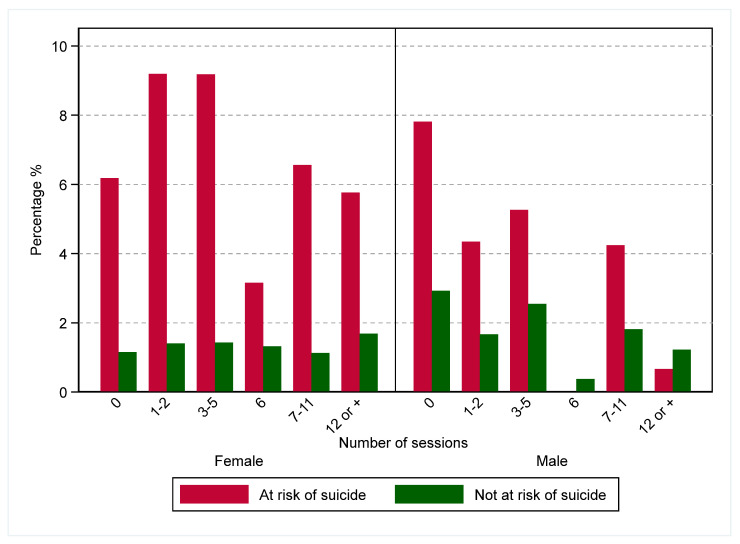
Hospital-treated self-harm within 12 months after the last service contact date by number of sessions, Western Sydney Primary Health Network, 2013–2018. Any hospital treated self-harm presentation (including cases of undetermined intent) within 12-months of the last service contact (or referral date for referrals that did not result in at least one follow-up treatment session) was presented as a proportion of primary mental health care service referrals over each session category.

There were 264 (2.2%) clients presenting with cases of hospital-treated self-harm who presented to a hospital setting within 12 months following the last service contact date or referral date (for those who never attended at least one treatment session). Overall, the rates of hospital-treated self-harm within 12 months were stable over the study period, and they were higher among females and younger age groups (those aged 18–24 years) (Figure 2 and Figure S1). Subsequent hospital-treated self-harm was also higher among those who presented to a primary health care setting at risk of suicide and those who attended fewer treatment sessions (<6 sessions) (Figure 3 and Figure S2).

**Figure 4:**
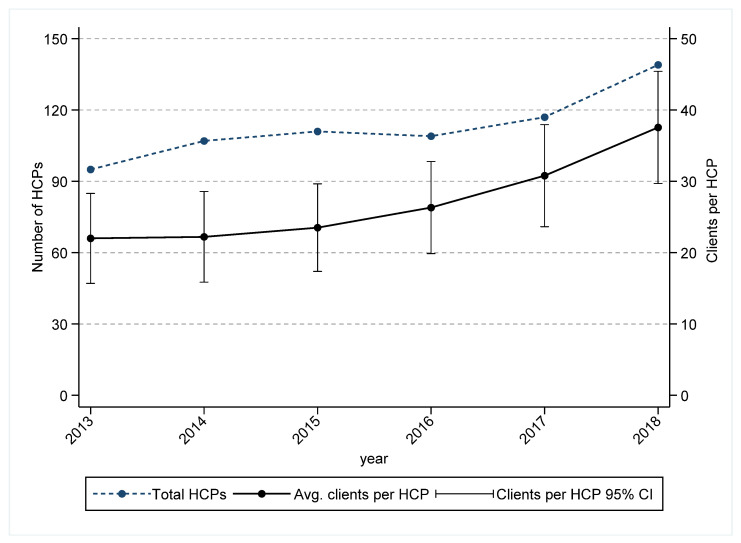
Trends in workforce capacity and patients per health care practitioner, Western Sydney Primary Health Network, 2013–2018. We excluded 2113 referrals, received from July 2016 to 31 December 2018, for 2082 clients (explained in the Supplementary Materials) due to the unavailability of HCP-identifiable information. However, de-identified HCP identifier information is available in the PMHC MDS for these excluded referrals, but there may be multiple HCP IDs for the same HCP given by different PMHC service provider organization levels if any HCP works in multiple PMHC service provider organizations.

**Figure 5:**
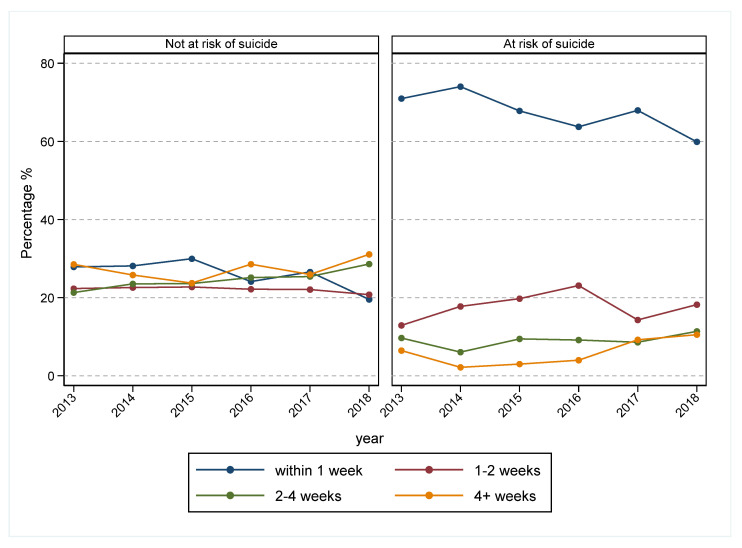
Trends in waiting time between referral and first treatment session by risk of suicide, Western Sydney Primary Health Network, 2013–2018. Waiting time was calculated based on the difference between the first session date and the referral date. Referrals of those who never attended at least one treatment session were excluded. Additionally, 1285 (8%) referrals were excluded due to the missing risk of suicide. A further 660 (4.5%) referrals, including those who were referred for low-intensity treatments, were excluded, as most of them accessed services via telephone and received services on the same day.

**Figure 6:**
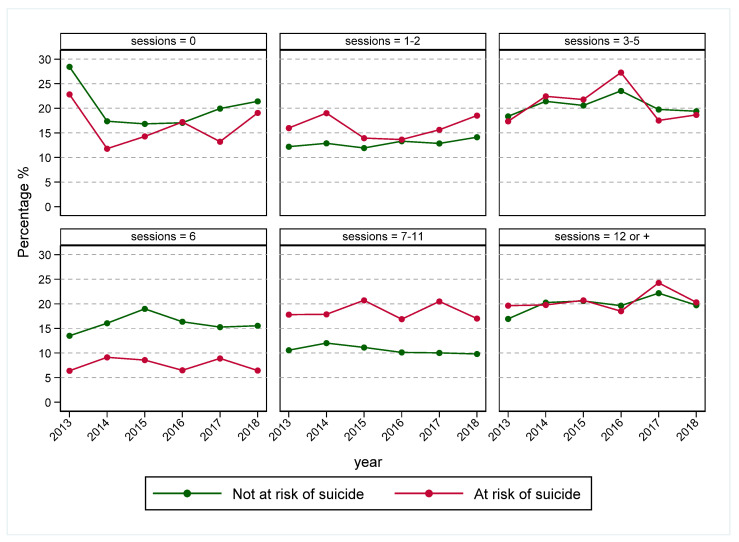
Trends in treatment non-attendance and sessions per referral by risk of suicide, Western Sydney Primary Health Network, 2013–2018. We excluded 2113 (10.9%) referrals. Of these referrals, 1784 (84%) comprised clients who were referred to low-intensity interventions, and this group was likely to have low session attendance per referral, especially those who accessed services via telephone, compared to other referrals. From the remaining 329 referrals, 70 had missing suicide flags and other data quality issues.

The total number of HCPs consistently increased over the period of 2013–2016 (Figure 4). However, the number of clients per HCP also increased from 2013 and increased more sharply in the period from 2016 (Figure 4). In comparison, waiting times for treatment initiation increased among those at risk of suicide (Figure 5), and the rates of treatment non-attendance and low attendance (0 and 1–2 sessions, respectively) also increased from 2016 (Figure 6).

## 4. Discussion

This study investigated the trends in PMHC service contacts and subsequent hospital-treated self-harm within 12 months following treatment over the period of 2013–2018 using key sociodemographic and service indicators. There was a sharp increase in the number of PMHC service referrals in the period after the establishment of PHNs in 2016, including an increase in the proportion of referred clients who were at risk of suicide. However, the trends in subsequent hospital-treated self-harm during the same period did not change substantially. Those who were at risk of suicide (that is, those who presented to primary health care settings in suicidal crisis or following discharge from a hospital setting) and who subsequently presented to a hospital setting following self-harm were more likely to either not attend PMHC services following a referral or to attend fewer services (compared to those not at risk of suicide). There was an increasing trend in the number of clients per HCPs (predominantly psychological services), despite an increase in the total number of HCPs in the WSPHN geographic catchment. There was also an increase in waiting times and treatment non-attendance over the study period.

Subsequent hospital-treated self-harm rates within the 12 months following treatment were similar to those of past studies that investigated subsequent self-harm presentations to hospital within 12 months following the first mental health presentation to a hospital setting [18]. However, the rates in the current study of subsequent self-harm within 12 months were lower than the rates of hospital-treated self-harm reported in a previous study that investigated the incidence of self-harm following discharge from psychiatric care [19], likely reflecting a lower severity of presenting mental disorders among PMHC clients than in inpatient settings. The finding of higher rates of hospital-treated self-harm among females and younger age groups is consistent with that of previous research [20], as are the overall rates of hospital-treated self-harm in Western Sydney reported elsewhere [21]. The variation in differences in hospital-treated self-harm between males and females aged 18–24 years reflects the stochastic variation from year to year due to the small number of self-harm incidences in the relatively small number of clients.

Subsequent hospital-treated self-harm was also higher among those at risk of suicide and who did not attend treatments (or who attended only a few sessions), similar to previous studies of those at risk of suicide, where treatment non-attendance among psychiatric patients was associated with an increased risk of acute care presentations [22] and subsequent suicidal behavior [10]. While previous research indicates ‘minimally adequate treatment’ as receiving at least six psychotherapy sessions of 30 min or over for those with mental disorders [23], a higher rate of hospital-treated self-harm among this group may reflect insufficient treatment to reduce suicidal thoughts and psychological distress. Additionally, our findings indicate that the risk of subsequent hospital-treated self-harm increased among those who received 7–11 and over 12 sessions. This group of clients may reflect those with more severe and ongoing mental health conditions (persistent thoughts of suicide and high psychological distress), those who may not have benefitted from the intensity of the treatment provided, and/or those who may have disengaged from treatments.

Our findings also indicate increasing trends of longer waiting times for treatment initiation for those who were referred to PMHC services in the recent period. Higher non-attendance and low attendance rates may be partly due to the increase in waiting times in the most recent period of the study, and timely access to treatment has been associated with improved treatment attendance and engagement in PMHC services [24,25]. Timely access to treatments has also been associated with improvements in mental health outcomes [26] and reductions in the risk of subsequent self-harm [27]. Despite the sharp increase in referrals since 2016, the current study also found that the workforce capacity in the region did not proportionally increase over the same period, and this may be associated with higher treatment non-attendance and low attendance rates.

There are a number of limitations to take into consideration when interpreting the results of the present study. Firstly, it is likely that hospital-treated self-harm in the current study is an under-enumeration of self-harm in the Western Sydney population, as only cases that presented to a hospital setting were included. It is also likely that some clients may have presented to hospital settings located outside the WSLHD catchment. Secondly, some clients who never attended or disengaged with treatment may have attended other mental health services not commissioned via PHNs (for example, mental health services within the Local Health District or other fee-for-service mental health services). Thirdly, referrals to, and from, low-intensity mental health services were also not captured where individual linkage information was not available, and the rates of hospital-treated self-harm may differ to those in the current study. However, acquiring person-identifiable information is difficult for those using low-intensity mental health services, such as helplines and other online platforms. Fourthly, it may be possible that HCPs have other clients outside the PMHC services considered in this study, and the actual clients per HCPs may differ from the estimates in the current study. Finally, full-time equivalent (FTE) proportions were not available for HCPs, and FTEs may vary by year for some HCPs.

## 5. Conclusions

Timely access to mental health treatment is important for improving mental health outcomes among those who access PMHC services. This study shows a substantial increase in PMHC service referrals following the establishment of PHNs in 2016, including an increase in the proportion of referred clients who were at risk of suicide. Those who were at risk of suicide and who subsequently presented to a hospital setting following self-harm were also more likely to either not attend PMHC services following a referral or to attend fewer services. Despite an increase in the total number of HCPs in the WSPHN geographic catchment, there has also been an increase in the number of clients per HCP, suggesting that the workforce capacity of PMHC services is not keeping pace with mental health demand.

## Figures and Tables

**Table 1:** Trends in primary mental health services and hospital-treated self-harm within 12 months of referral, Western Sydney Primary Health Network, 2013–2018.

Characteristics	2013	2014	2015	2016	2017	2018	Total
**Total referrals**	2262 (11.6)	2243 (11.5)	2420 (12.5)	2637 (13.6)	3544 (18.2)	6331 (32.6)	19,437 (100)
**Gender (gender is missing for 47 (0.24) referrals)**							
Male	849 (37.5)	940 (41.9)	993 (41.0)	1086 (41.2)	1372 (38.8)	2342 (37.2)	7582 (39.1)
Female	1412 (62.5)	1302 (58.1)	1427 (59.0)	1550 (58.8)	2165 (61.2)	3952 (62.8)	11,808 (60.9)
**Age (age is missing for 923 (4.75) referrals)**							
5–17	570 (25.3)	933 (41.6)	808 (33.4)	783 (29.7)	947 (27.5)	1122 (20.3)	5163 (27.9)
18–24	276 (12.3)	187 (8.3)	244 (10.1)	284 (10.8)	363 (10.5)	691 (12.5)	2045 (11.0)
25–44	827 (36.7)	595 (26.5)	726 (30.0)	789 (29.9)	1171 (34.0)	1881 (34.1)	5989 (32.3)
45–64	492 (21.8)	408 (18.2)	513 (21.2)	613 (23.3)	742 (21.6)	1452 (26.3)	4220 (22.8)
≥65	87 (3.9)	120 (5.3)	129 (5.3)	167 (6.3)	218 (6.3)	376 (6.8)	1097 (5.9)
**Socioeconomic status (socioeconomic status is missing for 1403 (7.22) referrals)**							
5 (Least disadvantaged)	197 (16.4)	396 (17.7)	430 (17.8)	395 (15.0)	550 (15.7)	952 (15.8)	2920 (16.2)
4	166 (13.8)	292 (13.0)	339 (14.0)	464 (17.6)	684 (19.5)	1154 (19.1)	3099 (17.2)
3	316 (26.3)	525 (23.4)	591 (24.4)	509 (19.3)	703 (20.0)	1491 (24.7)	4135 (22.9)
2	192 (16.0)	311 (13.9)	340 (14.1)	381 (14.5)	478 (13.6)	863 (14.3)	2565 (14.2)
1 (Most disadvantaged)	329 (27.4)	715 (31.9)	718 (29.7)	884 (33.6)	1092 (31.1)	1577 (26.1)	5315 (29.5)
**Diagnosis (diagnosis is missing for 1888 (9.71) referrals)**							
Anxiety disorders	324 (15.9)	434 (21.3)	434 (18.8)	425 (17.6)	854 (26.1)	1405 (25.6)	3876 (22.1)
Affective (mood) disorders	477 (23.4)	387 (19.0)	478 (20.7)	420 (17.4)	582 (17.8)	1075 (19.6)	3419 (19.5)
Anxiety and affective disorders	701 (34.4)	677 (33.2)	872 (37.7)	1034 (42.9)	1322 (40.5)	1933 (35.3)	6539 (37.3)
Substance use disorders	73 (3.6)	81 (4.0)	112 (4.8)	160 (6.6)	133 (4.1)	150 (2.7)	709 (4.0)
Other	465 (22.8)	462 (22.6)	415 (18.0)	369 (15.3)	377 (11.5)	918 (16.7)	3006 (17.1)
**Risk of suicide (risk of suicide is missing for 1373 (7.06) referrals)**							
No	2043 (90.3)	1980 (88.3)	2140 (88.4)	2329 (88.3)	2939 (88.4)	4258 (82.2)	15,689 (86.9)
Yes	219 (9.7)	263 (11.7)	280 (11.6)	308 (11.7)	385 (11.6)	920 (17.8)	2375 (13.1)
**Any medication use (any medication use is missing for 3108 (15.99) referrals)**							
No	1592 (70.4)	1590 (70.9)	1628 (67.3)	1730 (65.6)	1714 (59.2)	1961 (50.6)	10,215 (62.6)
Yes	670 (29.6)	653 (29.1)	790 (32.7)	906 (34.4)	1180 (40.8)	1915 (49.4)	6114 (37.4)
**Total referrals (linked data) ^a^**	1535 (12.7)	1302 (10.8)	1611 (13.3)	1852 (15.3)	2331 (19.3)	3440 (28.5)	12,071 (100)
**Intentional self-harm (undetermined included) ^b^**							
Within 365 days after LSD ^#^	28 (1.8)	23 (1.8)	37 (2.3)	30 (1.6)	60 (2.6)	86 (2.5)	264 (2.2)
Within 365 days after LSD ^$^	24 (2.1)	17 (1.6)	29 (2.1)	22 (1.4)	49 (2.6)	66 (2.4)	207 (2.1)
**Intentional self-harm (undetermined excluded) ^c^**							
Within 365 days after LSD ^#^	21 (1.4)	18 (1.4)	31 (1.9)	24 (1.3)	49 (2.1)	70 (2.0)	213 (1.8)
Within 365 days after LSD ^$^	18 (1.6)	13 (1.2)	25 (1.8)	19 (1.2)	40 (2.1)	54 (2.0)	169 (1.7)

Note: LSD—last service contact date; a—referrals with no client identification information (*n* = 2318) and clients below 18 years of age were excluded (explained in the Supplementary Materials). b—self-harm presentations related to undetermined intent were included and considered as self-harm, c—self-harm presentations with undetermined intent were excluded and not considered as self-harm. #—referral date was considered as LSD for referrals who never attended at least one service session to calculate 12-month self-harm. $—referrals who never attended at least one service session were excluded from the denominator.

## Data Availability

The data used in this study cannot be shared due to data custodian agreements, and access to the data has only been granted to the study team in accordance with ethical approvals. The codes and statistical programs can be provided by the authors on request.

## Supplementary Materials

The following supporting information can be downloaded at: https://www.mdpi.com/article/10.3390/ijerph19063382/s1, Figure S1: Trends in hospital treated self-harm within 12 months of last service contact by gender and age group, Western Sydney Primary Health Network, 2013-2018; Figure S2: Hospital treated self-harm within 12 months after the last service contact date by number of sessions, Western Sydney Primary Health Network, 2013–2018; a description about excluded participants; identification of Intentional self-harm in ED data; Table S1: Identification of self-harm incidents from ED dataset; data linkage method and quality of data linkage; Table S2: Matches and mismatches by probability cut-off according clerical review; Table S3: Matches and non-matches based on the clerical review and record linkage algorithm.
