# Supplementary material for: Trends in Primary Mental Health Care Service Use and Subsequent Self-Harm in Western Sydney Australia: Policy and Workforce Implications
Source: Int J Environ Res Public Health. 2022 Mar 13;19(6):3382. doi: 10.3390/ijerph19063382 (PMC8951314; doi:10.3390/ijerph19063382)
Supplement: Supplementary file 1 [file ijerph-19-03382-s001.zip › ijerph-1538109-supplementary.pdf]

Supplementary Material

Supplementary Figure S1: Trends in hospital treated self-harm within 12 months of last service contact by gender and age group, Western Sydney Primary Health Network, 2013-2018.*

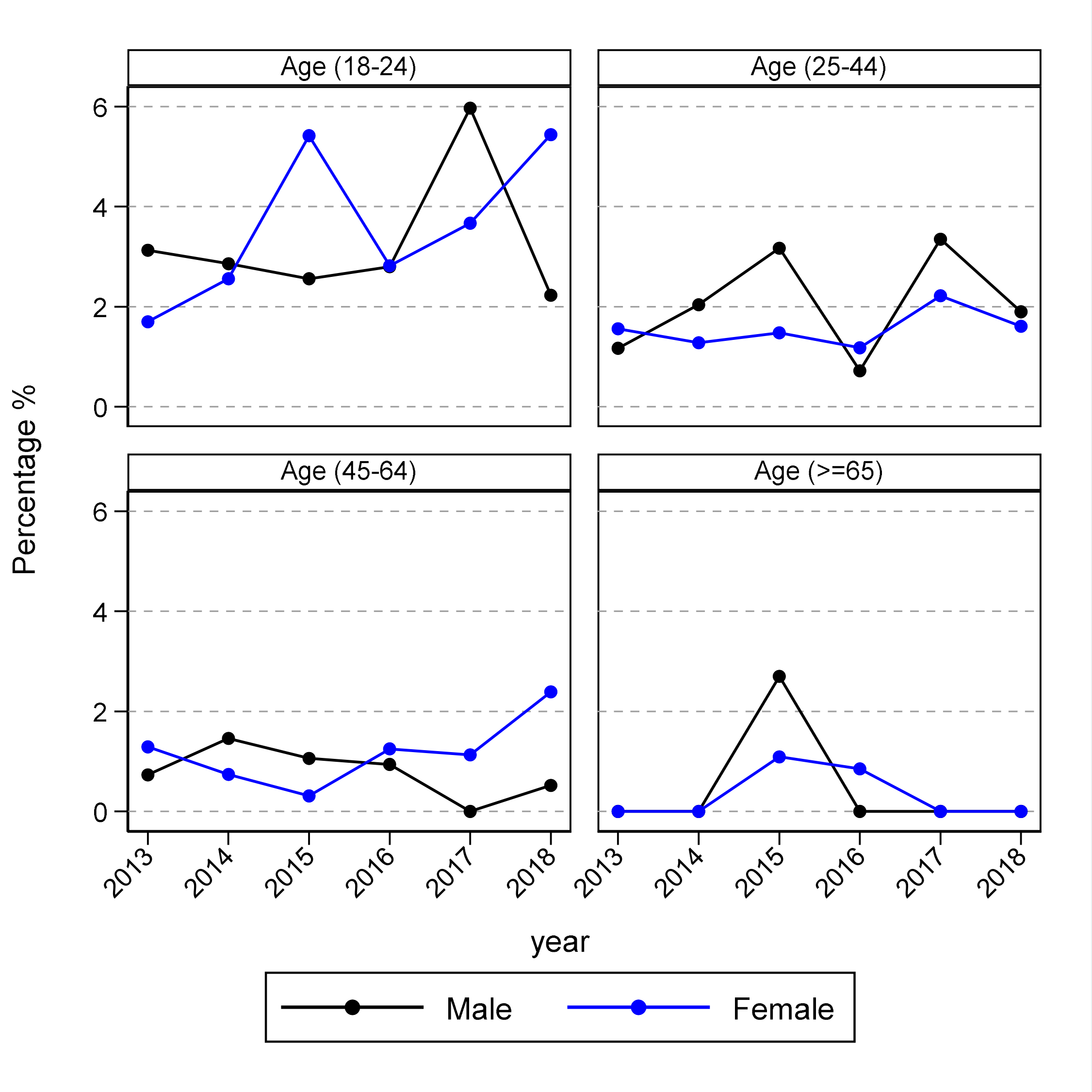

*Note: *Any hospital treated self-harm (excluding the cases of undetermined intent) presentation within 12-months of last service contact (or referral date for referrals that did not result in at least one follow-up treatment session) was presented as a proportion of primary mental health care service referrals in each year.*

Supplementary Figure S2: Hospital treated self-harm within 12 months after the last service contact date by number of sessions, Western Sydney Primary Health Network, 2013-2018.*

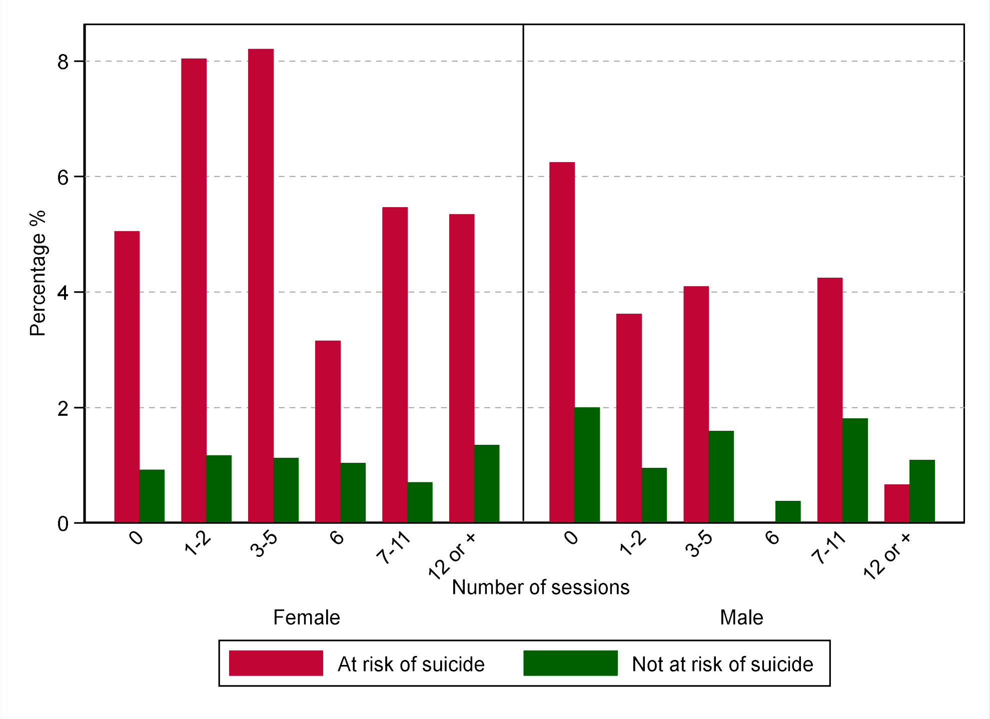

*Note: *Any hospital treated self-harm presentation (excluding cases of undetermined intent) within 12-months of last service contact (or referral date for referrals that did not result in at least one follow-up treatment session) was presented as a proportion of primary mental health care service referrals over each session category.*

Excluded Participants

The study included clients referred to PMHC services between 01 January 2013 and 31 December 2018. However, individual identifiable information (First name and last name) were not available for 2277 clients (2318 referrals). Of these 2277 clients, 181 referred in 2013 (9% of the referrals received in 2013) and 14 referred in 2014 (0.5 % of the referrals received in 2014), and the remaining 2113 referrals for 2082 clients were received after July 2016. The original referral forms of these 2082 clients did not directly receive by Western Sydney PHN and one of the representatives of each of the co-commissioning organisations, which provided services for these clients, have uploaded these data into the PMHC MDS and attach a copy of the MDS data in one of the Western Sydney PHN's internal database. These referrals include Statistical Linkage Key (SLK) 581 [1], derived based on patient name,

date of birth and gender. However, we could not simply locate the original SLKs for all of these referrals because some referrals were not consistently attached, although those were uploaded into PMHC MDS, in the earlier period of 2017-18. Data we extract from the PMHC MDS does not include this original SLK-581 and it is converted into an unidentified 32 character long hashed SLK. However, nearly 45% of the clients did not record an accurate date of birth (most likely among clients who reach mental health services via telephone). Therefore, we could not use about half of these referrals even if these referrals would have had the original SLKs.

Referrals those were excluded predominantly for low-intensity mental health treatments (1784, 84.43%), followed by other mental health treatments (127, 6.01%), and psychological therapy (95, 4.50%). Referrals those were included predominantly for psychological therapy (12221, 70.58), followed by child and youth specific (2971, 17.16%) and other (1690, 9.76%). Further, data for those aged <18 years were not available for this analysis, given current Institutional Ethics Committee and Data Custodian approvals..

DATA LINKAGE

Data Linkage Algorithm and software

Data linkage was conducted using R software (version 3.6.0 [2]), and used the 'RecordLinkage' package developed by Sariyar M & Borg A [3]. This package includes three main data linkage algorithms (i) the Fellegi-Sunter method, (ii) the Expectation maximization method and (iii) the Contiero method [4]. We used the Contiero method for the data linkage as it is more straightforward to implement, and can

achieve a higher sensitivity and specificity based on previous research [4]. Although, previous research has also shown similar performance for each of these three methods [5, 6].

Linkage variables of the currant data linkage study

The current study included four linkage variables; (i) first name, (ii) last name, (iii) date of birth, and (iv) gender. First name and last name are string variables, and date of birth was divided into birth year, birth month, and day of birth.

Data linkage process

The Jaro-Winkler similarity measure [7] was employed to compute the similarity between two string pairs, while the binary exact method was used for numeric variables (which gives 1 for a numeric pair match, and 0 for a numeric pair non-match)

Other personal information such as patient address, mobile number and Medicare number were not available in the PMHC dataset, although this information may change over time. However, the current linkage used client postcode as an additional measure to support the decision in identifying a pair as a match or non-match (see below). The linkage variables used in this study were considered less likely to change over time, although it is acknowledged that for this is not the case in all instances. For example, surname of female patients may change following marriage or divorce. In these instances, this is the most likely reason for a non-match for the same individual if they present to one service prior to a surname change and then to another service after a surname change. These limitations are, however, common in large administrative data sources and manual inspection (see below) following data linkage can identify potential false positive and false negative matches.

Blocking variables for the current data linkage study

In the absence of a blocking variable/s, probabilistic record linkage may be time intensive for large data sources, and time consuming to identify a matching threshold in the presence of a large number of

matching pairs. For example, the current study would have created 10,071 (number of unique clients included in PMHC data sources) x 695,786 (number of unique clients included in hospital data sources) record pairs if blocking variables were not specified. Therefore, this linkage study used gender and date of birth as blocking variables in the following vector form ((gender, birth year, birth month), (gender, birth year, day of birth), (gender, birth month, day of birth)). Every record pair following the above blocking methods contains same gender as well as same birth year and birth month or same birth month and day of birth or same birth year and day of birth. For pairs with lower probability weights were re-run by applying date of birth alone as a blocking variable in the form of ((birth year, birth month), (birth year, day of birth), (birth month, day of birth)).

Selecting a linkage threshold

Following the probabilistic data linkage, matched pairs with probability weights greater than 0.7 was selected for clerical review. The probability of similarity ranges from 0 to 1, and a high probability reflects a high similarity of a given pair. There were 124,864 total linked pairs for the 10,027 individuals in the PMHC dataset. Record pairs with maximum probability weights considered as matched pairs (for example, if individual A1 in the PMHC dataset matches with individuals A2 and A3 in the hospital dataset with probability weights 0.78 and 0.92, respectively, then we consider A1 and A3 as the matched pair). The following rules for clerical review were defined in order to select an optimal cut-off to maximise the sensitivity and specificity of the data linkage.

Clerical review and criteria to identify true matches and non-matches

1. If the date of birth exactly matches, and two names match either exactly or nearly, then the pair is considered as a true matched pair.

2 If the date of birth does not match exactly, but first name and family name match either exactly or nearly, then the pair is considered a true matched pair only if the postcode also matches exactly. In instances where postcode did not match, this was considered a potential matched pair.

3 If the date of birth matches exactly and one part of the name matches either exactly or nearly, then the pair is considered a potential match only if the postcode also matches exactly, and a non-match if the postcode does not match exactly.

4 If the date of birth does not match exactly and one part of the name matches either exactly or nearly, then the pair is not considered as a matched pair.

Supplementary Table S1: Matches and mismatches by probability cut-off according clerical review.

Probability threshold	DOB match exactly		At least one name matches nearly or exactly		Two names match exactly or nearly		Potential match	Exact match	Total matches
0.70-0.80#	Yes	72	Yes	3	Yes	0	1	1	2
	No	128	Yes	64	Yes	0	0	0	0
0.80-0.85$	Yes	57	Yes	35	Yes	2	12	2	14
	No	43	Yes	42	Yes	2	0	0	0
0.85-0.86	Yes	27	Yes	24	Yes	0	5	0	5
	No	15	Yes	15	Yes	4	1	3	4
0.86-0.87	Yes	34	Yes	29	Yes	3	5	3	8
	No	78	Yes	78	Yes	73	25	47	72
0.87-0.88	Yes	17	Yes	15	Yes	3	2	3	5
	No	8	Yes	8	Yes	5	2	3	5
0.88-0.89	Yes	15	Yes	14	Yes	2	5	2	7
	No	8	Yes	8	Yes	4	1	3	4
0.89-0.90	Yes	16	Yes	16	Yes	2	6	2	8
	No	7	Yes	7	Yes	7	2	5	7
0.90-0.91	Yes	8	Yes	8	Yes	4	2	4	6
	No	101	Yes	101	Yes	101	35	66	101
0.91-0.95	Yes	79	Yes	79	Yes	64	8	64	72
	No	67	Yes	67	Yes	67	14	53	67
0.95-0.99	Yes	697	Yes	697	Yes	697	0	697	697
	No	0	Yes	0	Yes	0	0	0	0
1.00	Yes	6274	Yes	6274	Yes	6274	0	6274	6274
	No	0	Yes	6274	Yes	0	0	0	0

Note: # - there were 1988 linked individuals included in the 0.7-0.8 probability threshold and we randomly selected 200 individuals for clerical review. $ - there were 588 linked individuals included in the 0.8-0.85 probability threshold and we randomly selected 100 individuals for clerical review

Further, we observed that postcodes were more likely to be exactly similar among record pairs with non-exact date of birth, and exact, or near exact, first name and last name than record pairs with exactly same date of birth and mismatched first name or last name. This ensures the appropriateness of our criteria to identify matches and non-matches by clerical review.

Quality of record linkage

Linkage quality is a measure of the ability of the algorithm to identify a true match as a match and a true non-match as a non-match. Linkage quality of the current linkage activity is presented in terms of sensitivity and specificity. Sensitivity refer to the percentage of true matches identified by the algorithm, while specificity refers to the percentage of true non-matches identified for a given probability threshold. According to the probability distribution, we considered probability cot-off as 0.85. Sensitivity and specificity of the current linkage study was calculated as follows.

Supplementary Table S2: Matches and non-matches based on the clerical review and record linkage algorithm.

	Clerical review match	Clerical review non-match
Record linkage match	7342 (a)	109 (b)
Record linkage non-match	123 (c)	2453 (d)
Total	7465 (a+c)	2562 (b+d)

Sensitivity = $\frac{a}{a+c} = \frac{7342}{7342+123}$ = 98.37%

Specificity = $\frac{d}{b+d} = \frac{2453}{2453+109}$ = 95.74%

Since we have only reviewed a 200 and 100 random samples for 1988 and 588 total pairs of probability weight 0.70-0.80 and 0.80-0.85, respectively, it is likely the above sensitivity and specificity estimates may slightly differ from actual values. However, these differences are likely negligible and actual sensitivity and specificity values should exceed 95%.

Intentional self-harm in ED data

ED dataset collects diagnoses in SNOMED diagnoses codes and another free text diagnosis field called 'presenting problem'. We manually went through each single diagnosis in each of the SNOMED (2,907 different diagnoses) diagnosis and 'presenting problem' (1,515 different presenting problems) to identify incidents of self-harm and suicidal ideation. According to the SNOMED diagnosis and 'presenting problem', we divide self-harm incidents mainly into three categories; (i) intentional self-harm, (ii) accidental self-harm and (iii) self-harm with undetermined intent. Final self-harm presentation identification is explained in the table below.

Supplementary Table S3: Identification of self-harm incidents from ED dataset.

SNOMED diagnosis	Presenting problem						
	None	SH	Accidental SH	Undetermined SH	Suicidal ideation	Missing	Total
None	16,105 (0)	234 (1)	7 (0)	102 (2)	731 (0)	5 (0)	17,184
SH	107 (1)	261 (1)	2 (1)	110 (1)	106 (1)	0	586
Accidental SH	19 (0)	2 (1)	6 (0)	21 (0)	1 (1)	0	49
Undetermined SH	199 (2)	77 (1)	2 (0)	287 (2)	72 (1)	0	637
Suicidal ideation	252 (0)	152 (1)	1 (1)	32 (1)	876 (0)	0	1,313
Missing	1,352 (0)	32 (1)	0	4 (2)	55 (0)	2 (0)	1,445
Total	18,034	758	18	556	1,841	7	21,214

Note: 0- Incidents neither considered as intentional self-harm nor potential self-harm, 1 – incidents considered as intentional self-harm; 2- incidents considered as potential intentional self-harm.

It is likely that there will be intentional self-harm presentations among the presentations we identified as undetermined self-harm. However, we may identify some of them if they had admitted to the hospital based on ICD 10 codes, but there may be a group did not end up in admitting to the hospital. Therefore, we ran the same analysis separately for (i) intentional self-harm and (ii) intentional self-harm or undetermined self-harm.

REFERENCES

1. Australian Institute of Health and Welfare. SLK-581 Guide for use. 2016. https://www.aihw.gov.au/getmedia/e1d4d462-8efa-4efa-8831-fa84d6f5d8d9/aodts-nmds-2016-17-SLK-581-guide.pdf.aspx. (accessed oct2021).

2. R Development Core Team. R: a language and environment for statistical computing Vienna, Austria: R Foundation for Statistical Computing. 2011.

3. Sariyar M, Borg A. Package 'RecordLinkage'. 2020.

4. Contiero P, Tittarelli A, Tagliabue G, et al. The EpiLink record linkage software. Methods of Information in Medicine. 2005;44(01):66-71.

5. Morgan AS, Marlow N, Costeloe K, Draper ES. Investigating increased admissions to neonatal intensive care in England between 1995 and 2006: data linkage study using Hospital Episode Statistics. BMC Medical Research Methodology. 2016;16(1):1-11.

6. Karr AF, Taylor MT, West SL, et al. Comparing record linkage software programs and algorithms using real-world data. PloS One. 2019;14(9):e0221459.

7. Winkler WE. String Comparator Metrics and Enhanced Decision Rules in the Fellegi-Sunter Model of Record Linkage. 1990.
